# Cardiac Implantable Device Infection Surveillance Algorithm

**DOI:** 10.1001/jamanetworkopen.2025.14079

**Published:** 2025-06-05

**Authors:** Hillary J. Mull, Samuel W. Golenbock, Dipandita Basnet Thapa, Marlena H. Shin, Kimberly L. Harvey, Rebecca P. Lamkin, Judith Strymish, Westyn Branch-Elliman

**Affiliations:** 1 Center for Health Optimization and Implementation Research, Veterans Affairs (VA) Boston Healthcare System, Boston, Massachusetts; 2 Department of Surgery, Chobanian & Avedisian School of Medicine, Boston University, Boston, Massachusetts; 3 Department of Medicine, Section of Infectious Diseases, VA Boston Healthcare System, Boston, Massachusetts; 4 Department of Medicine, Harvard Medical School, Boston, Massachusetts; 5 Department of Medicine, Greater Los Angeles VA Healthcare System, Los Angeles, California; 6 University of California Los Angeles David Geffen School of Medicine, Los Angeles

## Abstract

This cohort study describes changes to an existing tool for detecting infections related to placement of an cardiovascular implantable electronic device.

## Introduction

Cardiovascular implantable electronic device (CIED) procedure–related infections have high morbidity and mortality.^1^ An electronically augmented infection monitoring system was developed and validated to support identification of CIED infections.^2^ This novel tool identified possible infections based on structured data fields and free-text clinical notes using text-string searches in the Veterans Health Administration (VHA) electronic health records (EHRs).^3^ Since 2022, the tool has been operational through manual review of procedures (hereafter cases) with high estimated infection probability. We assessed the performance of the original CIED-related infection detection algorithm with clinical implementation data to identify improvement opportunities before national dissemination.^4^

## Methods

The Veterans Affairs (VA) Boston Healthcare System Institutional Review Board approved this cohort study and waived informed consent because it was not human participant research. We followed the STROBE reporting guideline.

Following methods for developing the original algorithm,^2^ we identified new EHR flags from VHA Community Care (CC) datasets and modified original flags using comorbidity software, diagnoses, antibiotic administration records, microbiological specimen orders, and clinical notes in the VA Corporate Data Warehouse to create a multivariate logistic regression model estimating CIED-related infection up to 90 days after the procedure (eMethods, eFigure, and eTable in Supplement 1). To refine the algorithm, we used medical records–reviewed CIED cases with a high probability of infection from the active surveillance period (June 1, 2021-April 30, 2023). Important flags and estimated probabilities from the original algorithm were compared with estimated probabilities from the refined algorithm to increase positive predictive validity (PPV) of the algorithm without increasing the number of cases flagged for review (flag rate). New CIED cases from the surveillance period with a possible infection were reviewed to measure PPV of the refined algorithm. All analyses were completed using SAS (SAS Institute).

## Results

There were 12 927 new CIED cases in the surveillance period. Manual review of flagged cases found 98 of 334 cases (29.3%) had true infections. In bivariate analyses, changes to improve algorithm case ascertainment without increasing review burden included (1) updating the definition of index cases as new CIED procedures in the VHA with no prior procedure in the VHA or CC in the prior 90 days and (2) changing the timing of infection flags to start 0 to 3 days, instead of 3 to 6 days, after CIED procedure (eg, microbiological order: 0-90 vs 3-90 days) (Table 1). Flags for history of CIED-related infection were not associated with infection.

**Table 1.** Characteristics of Potential CIED-Related Infection in Index Cases, Cases With Medical Record Review, and Cases With Infection

Characteristic	Flag rate in index cases, No. (%) (n = 12 927)^a^	Medical record reviewed		
		Total, No. (%) (n = 334)	Infected, No. (%) (n = 98)	*P* value^b^
Procedure outcomes				
New: died <30 d postprocedure^c^	117 (0.9)	8 (2.4)	0	.07
Died <90 d postprocedure^d^	338 (2.6)	32 (9.6)	7 (7.1)	.33
Billing: diagnosis code				
Emergent problem	1598 (12.4)	37 (11.1)	12 (12.2)	.66
*ICD-10* code CIED infection 3-90 d postprocedure – VHA^d^	387 (3.0)	140 (41.9)	79 (80.6)	<.001
*ICD-10* code for SSI infection only 3-90 d postprocedure – VHA^d^	88 (0.7)	35 (10.5)	9 (9.2)	.62
New: *ICD-10* code CIED infection 0-90 d postprocedure – VHA^c^	394 (3.1)	144 (43.1)	82 (83.7)	<.001
New: *ICD-10* code SSI infection only 0-90 d postprocedure – VHA^c^	90 (0.7)	34 (10.2)	8 (8.2)	.43
New: *ICD-10* code CIED infection 0-90 d postprocedure – Community Care^c^^,^^e^	95 (0.7)	33 (9.9)	27 (27.55)	<.001
New: *ICD-10* code CIED infection 3-90 d preprocedure – VHA^c^	218 (1.7)	21 (6.3)	3 (3.1)	.12
New: *ICD-10* code CIED infection 3-90 d preprocedure – Community Care^c^^,^^e^	32 (0.3)	4 (1.2)	1 (1.0)	.85
Billing: procedure code				
New: any CIED removal procedure 3-90 d postprocedure – VHA^c^	411 (3.2)	60 (18.0)	37 (37.8)	<.001
New: any CIED removal procedure 3-90 d postprocedure – Community Care^c^^,^^e^	53 (0.4)	14 (4.2)	13 (13.3)	<.001
New: any CIED procedures 91-365 d preprocedure – VHA^c^	202 (1.6)	7 (2.1)	2 (2.0)	.96
New: any CIED procedures 91-365 d preprocedure – Community Care^c^^,^^e^	51 (0.4)	1 (0.3)	0	.52
Pharmacy order				
No antibiotic with duration ≥3 consecutive d, ordered 6-90 d postprocedure^d^	10 751 (83.2)	93 (27.8)	23 (23.5)	.25
Any antibiotic or drug class with duration ≥3 consecutive d, ordered 6-90 d postprocedure	2176 (16.8)	241 (72.2)	75 (76.5)	.25
Antibiotic typically used to treat *Staphylococci*^d^	1832 (14.2)	225 (67.4)	73 (74.5)	.08
Antibiotic not typically used to treat *Staphylococci*^d^	344 (2.7)	16 (4.8)	2 (2.0)	.13
New: no antibiotic with duration ≥3 consecutive d, ordered 3-90 d postprocedure^c^	10 519 (81.4)	65 (19.5)	8 (8.2)	<.001
New: any antibiotic or drug class with duration ≥3 consecutive d, ordered 3-90 d postprocedure^c^	2408 (18.6)	269 (80.5)	90 (91.8)	<.001
Antibiotic typically used to treat *Staphylococci*	2239 (17.3)	265 (79.3)	90 (91.8)	<.001
Antibiotic not typically used to treat *Staphylococci*	169 (1.3)	4 (1.2)	0	
Laboratory orders				
Cardiac specimens from bacteriological reports with positive cultures with antibiotic susceptibility testing performed 3-90 d postprocedure^d^	126 (1.0)	50 (15.0)	30 (30.6)	<.001
New: cardiac specimens from bacteriological reports with positive cultures with antibiotic susceptibility testing performed 0-90 d postprocedure^c^	126 (1.0)	50 (15.0)	30 (30.6)	<.001
New: blood specimens from bacteriological reports with positive cultures with antibiotic susceptibility testing performed 0-90 d postprocedure^c^	1072 (8.3)	174 (52.1)	83 (84.7)	<.001
New: miscellaneous (eg, wound, abscess) specimens from bacteriological reports with positive cultures with antibiotic susceptibility testing 0-90 d postprocedure^c^	247 (1.9)	65 (19.5)	43 (43.9)	<.001
Clinical note text				
Keyword search for *infection* 3-90 d postprocedure^d^	1043 (8.1)	285 (85.3)	92 (93.9)	.004
New: keyword search for *CIED infection* 0-90 d postprocedure^c^	1217 (9.4)	264 (79.0)	93 (94.9)	<.001
New: keyword search for *abscess* 0-90 d postprocedure^c^	41 (0.3)	18 (5.4)	8 (8.2)	.15
Keyword search for *infection* 3-90 d preprocedure^d^	462 (3.6)	15 (4.5)	8 (8.2)	.04
New: keyword search for *CIED infection* 1-90 d preprocedure^c^	647 (5.0)	42 (12.6)	13 (13.3)	.81
New: keyword search for *abscess* 1-90 d preprocedure^c^	21 (0.2)	2 (0.6)	1 (1.0)	.52

Abbreviations: CIED, cardiovascular implantable electronic device; *ICD-10*, *International Statistical Classification of Diseases and Related Health Problems, Tenth Revision*; SSI, surgical site infection; VHA, Veterans Health Administration.

^a^ Index cases are CIED procedures in the VHA from June 1, 2021, to April 30, 2023, with no CIED procedure in prior 1 to 90 days.

^b^ χ^2^ tests of significance were completed for flags and infection in the sample that was manually reviewed, with α = .05.

^c^ *New* flags are those tested for the refined algorithm and indicate specific changes from the original flags.

^d^ Procedure characteristics used as flags in original CIED-related infection detection algorithm.

^e^ Community Care data include claims for procedures and visits or admissions with diagnostic data. Pharmacy and laboratory data are not available in Community Care claims.

The refined algorithm included revised flags based on timing (eg, antibiotic prescribed for ≥3 days for *Staphylococci*: odds ratio [OR], 1.79; 95% CI, 0.51-6.26), revised text-based clinical note flags of new or recent CIED-related infection (OR, 4.07; 95% CI, 1.37-12.10), and new CIED case and CC diagnosis flags (Table 2). Specific comorbidities, 90-day mortality, and history of infection were no longer associated with new infection in the refined algorithm.

**Table 2.** Logistic Regression of CIED-Related Infection Detection in Original and Refined Algorithms

Model variable	Refined algorithm, OR (95% CI)
Procedure outcomes	
Died <90 d postprocedure	Original algorithm only^a^
Comorbidity	
Congestive heart failure	Original algorithm only^a^
Solid tumor without metastasis	Original algorithm only^a^
Billing data	
*ICD-10* code for CIED infection in VHA 0-90 d postprocedure	11.52 (4.76-27.86)
*ICD-10* code for SSI 0-90 d postprocedure	4.50 (1.31-15.41)
*ICD-10* code for CIED infection in CC 0-90 d postprocedure^b^	3.13 (0.97-10.11)
History of *ICD-10* code for CIED infection in VHA or CC^b^	0.26 (0.05-1.29)
*CPT* code for new CIED procedure in VHA or CC postprocedure^b^	2.20 (0.90-5.38)
Pharmacy data	
Antibiotic ≥3 d to treat *Staphylococci* ordered 3-90 d postprocedure	1.79 (0.51-6.26)
Laboratory data	
Micro order: cardiac 0-90 d postprocedure	1.72 (0.66-4.43)
Micro order: blood 0-90 d postprocedure^b^	2.41 (1.07-5.46)
Micro order: miscellaneous 0-90 d postprocedure^b^	2.87 (1.25-6.62)
Text-based clinical note^c^	
Diagnosis of CIED infection 0-90 d postprocedure	4.07 (1.37-12.10)
History of infection	Original algorithm only^a^

Abbreviations: CC, Community Care; CIED, cardiovascular implantable electronic device; *CPT*, *Current Procedural Terminology*; *ICD-10*, *International Statistical Classification of Diseases and Related Health Problems, Tenth Revision*; OR, odds ratio; SSI, surgical site infection; VHA, Veterans Health Administration.

^a^ Indicates that the flag appeared in the original algorithm only and was not significant in the refined algorithm.

^b^ Indicates a new type of variable in the refined algorithm that was not in the original algorithm. All variables in the refined algorithms have been modified slightly by timing or coding, as described in the eMethods in Supplement 1.

^c^ The *C* statistic, a measure of accuracy and model fit, was 0.909.

Compared with the original algorithm, the refined algorithm had improved case ascertainment (PPV, 29.3% [98 of 334] vs 56.7% [90 of 159]) and a similar flag rate (2.9% [370 of 12 927] vs 2.8% [364 of 12 927]). Of 98 infections in the reviewed sample, 90 (91.8%) were flagged by both algorithms. The refined algorithm identified 205 CIED cases from the surveillance period that were not flagged in the original algorithm. A random reviewed sample of new cases yielded PPV of 74.0% (37 of 50).

## Discussion

During implementation of the CIED-related infection surveillance tool, we identified opportunities to improve case ascertainment. The original algorithm excluded CC; thus, adding CC flags identified previously undetected infections.^5^ Study limitations include follow-up being more complete in VHA than other settings; the VHA population not being representative of the US population, which may skew findings; and the tool being reliant on manual review.

The biggest challenge of moving from development to clinical implementation was incorporating time and timing of infections relative to CIED procedure. Lessons learned about the need for adaptation may be helpful for leveraging EHR-based informatics tools to support surveillance activities.^6^

## Notes

Supplement 1. **eMethods.** Additional Details on Algorithm Development Methodology **eFigure.** Potential Changes to Redefine Index CIED Procedures and Modify Flags for CIED Infection **eTable.** Comparison of Changes in Timing for CIED Infection Prediction Flags From Original and Refined Algorithm
eReferences

Supplement 2.
Data Sharing Statement
