# Supplementary material for: Cardiac Implantable Device Infection Surveillance Algorithm
Source: JAMA Netw Open. 2025 Jun 5;8(6):e2514079. doi: 10.1001/jamanetworkopen.2025.14079 (PMC12142443; doi:10.1001/jamanetworkopen.2025.14079)
Supplement: Supplement 1. — eMethods. Additional Details on Algorithm Development Methodology eFigure. Potential Changes to Redefine Index CIED Procedures and Modify Flags for CIED Infection eTable. Comparison of Changes in Timing for CIED Infection Prediction Flags From Original and Refined Algorithm eReferences [file jamanetwopen-e2514079-s001.pdf]

Supplemental Online Content

Mull HJ, Golenbock SW, Basnet Thapa D, et al. Cardiac implantable device infection surveillance algorithm. *JAMA Netw Open*. 2025;8(6):e2514079. doi:10.1001/jamanetworkopen.2025.14079

eMethods. Additional Details on Algorithm Development Methodology

eFigure. Potential Changes to Redefine Index CIED Procedures and Modify Flags for CIED Infection

eTable. Comparison of Changes in Timing for CIED Infection Prediction Flags From Original and Refined Algorithm

eReferences

This supplemental material has been provided by the authors to give readers additional information about their work.

Methods. Additional Details on Algorithm Development Methodology

Data Sources and Study Population:

The study population included CIED procedures performed in the VA between June 2021 – April 2023. We refined our algorithm in previously identified CIED procedures selected for infection surveillance; each case had a predicted probability of an infection >10%. In addition to the infection flags and patient demographic data in the original algorithm extracted from the VA Corporate Data Warehouse (CDW),[1,2] we added VA paid community care (CC) claims from the Office of Integrated Veteran Care Consolidated Data Set.[3] Additional changes to the dataset included the revised comorbidity software from Agency for Healthcare Research and Quality (AHRQ).[4]

Definition of CIED Infection:

The purpose of our CIED infection surveillance system was to detect CIED infections within 90 days of placement (e.g., procedure-related infections, rather than secondary infections); however, we did not exclude infections present at the time of procedure unless the infection could be attributed to a CIED procedure 90 days prior.[2,5] CIED infections were classified into: pocket infections, systemic infections/endocarditis, and lead infections. Infection status was determined based on a minimum of two criteria among the following: physician documentation of "infection" with associated antimicrobial treatment, documentation of at least two symptoms consistent with a CIED infection (e.g., fever, redness, warmth, or purulent drainage), imaging findings consistent with a cardiac device infection or endocarditis (e.g., echocardiogram results) microbiologic testing (e.g., blood cultures, wound cultures), laboratory tests and/or antibiotic treatment up to 90 days post-procedure. We did not consider superficial cellulitis in other locations or stitch abscesses to be CIED infections.[6]

Algorithm Refinement:

Our first approach to refining the surveillance process was to more reliably exclude CIED procedures that were due to treatment of a recent infection, suggesting that the flag was incorrectly identifying the *timing* of the infection and thereby attributing it to the incorrect index procedure (in other words, identifying procedures that were performed for the management of an infection, rather than procedures where the infection occurred after the procedure). To improve upon the algorithm's precision with regard to timing, we updated the definition of index CIED procedures as new procedures in the VA with no prior CIED procedure in the VA or CC in the prior 90 days (e**Figure**). All of the flags from our original algorithm were retained in our dataset; however, these were modified to identify CIED infections present-on-admission given our revised definition (e**Table**). In our original algorithm, flag timing was 6-90 days after the CIED procedure. The revised algorithm used 0-90 days for ICD-10 code diagnoses of either a surgical site infection or cardiac device infection, an order of a cardiac, blood, or abscess microbiology specimen, and a text-based clinical note flags diagnosis of a new CIED infection. The original algorithm also used a text-based clinical note flag of a historical diagnosis of a CIED infection from 3-90 days prior to the index CIED procedure that was changed to -1-90 days in the refined algorithm. Similarly, the first iteration of the algorithm included flags for any antibiotic prescribed for ≥ 3 days and antibiotics typically used to treat *Staphylococcal* infections in a 6-90 day window; the revised algorithm expanded the window to 3-90 days. We retained a flag for emergent procedure and death within 90 days.

Next, we created new flags to rule out historical CIED infections and identify incident infections from VA and CC billing data. We identified historical CIED procedures using CPT codes from VA and CC data in the year before the index date (365 to 91 days before the index CIED procedure). We also collected historical data on CIED infection ICD-10 codes in the 90 days before the index CIED event. Using more detailed text parsing we created four additional text-based clinical note flags for a history of or new CIED-specific infection or abscess infections. We developed a new flag for death in 30 days, and added antibiotic data from the intravenous drug administration data in the CDW to ensure we were identifying

© 2025 Mull HJ et al. *JAMA Network Open*

all instances of extended antibiotic use in the 90 days after the index procedure. Lastly, we updated comorbidity flags using the new AHRQ Elixhauser Comorbidity Software refined for ICD-10 codes.[4]

We used the manually reviewed cases from real-time CIED infection surveillance to test the predictive utility of the refined algorithm. The various flags for new and historic CIED infections were added to a Random Forest Model machine learning algorithm with maximum trees set at a maximum of 1000, leaf size set at 6, and node size set at 100,000.[7] Variables with a non-negative out-of-bag Gini values in the loss reduction variable importance table were used to estimate a mixed effects multivariate logistic maximum likelihood model (Proc Glimmix with the Gauss-Hermite quadrature estimation method, random effects to account for variance, and the Newton Raphson optimization technique) to assign a new predicted probability of infection to each CIED procedure.[8]

Analysis:

Our analyses included bivariate comparisons of original and refined CIED infection flags in the overall sample of CIED cases without a 90-day prior CIED procedure and in the real-time chart reviewed data with infection outcomes (chi-square test and t-tests as appropriate, alpha set at 0.005). We also calculated threshold for positive predictive validity (PPV), negative predictive validity (NPV), sensitivity, and specificity for our refined predictive algorithm score and the c-statistic/receiver operating curve value from the final multivariate model to measure accuracy and model fit. By applying the refined algorithm score to the overall CIED dataset, we determined the flag rate of the final refined algorithm and compared the number of cases reviewed in the real-time surveillance process versus the number of cases that would be flagged for review by the refined algorithm. In the event the refined algorithm identified CIED cases with a score predicting infection that were **not** identified by the original algorithm, we randomly sampled 50 cases for chart review and measured PPV. All analyses were completed using SAS software.[9]

© 2025 Mull HJ et al. *JAMA Network Open*

eFigure. Potential Changes to Redefine Index CIED Procedures and Modify Flags for CIED Infection [a]

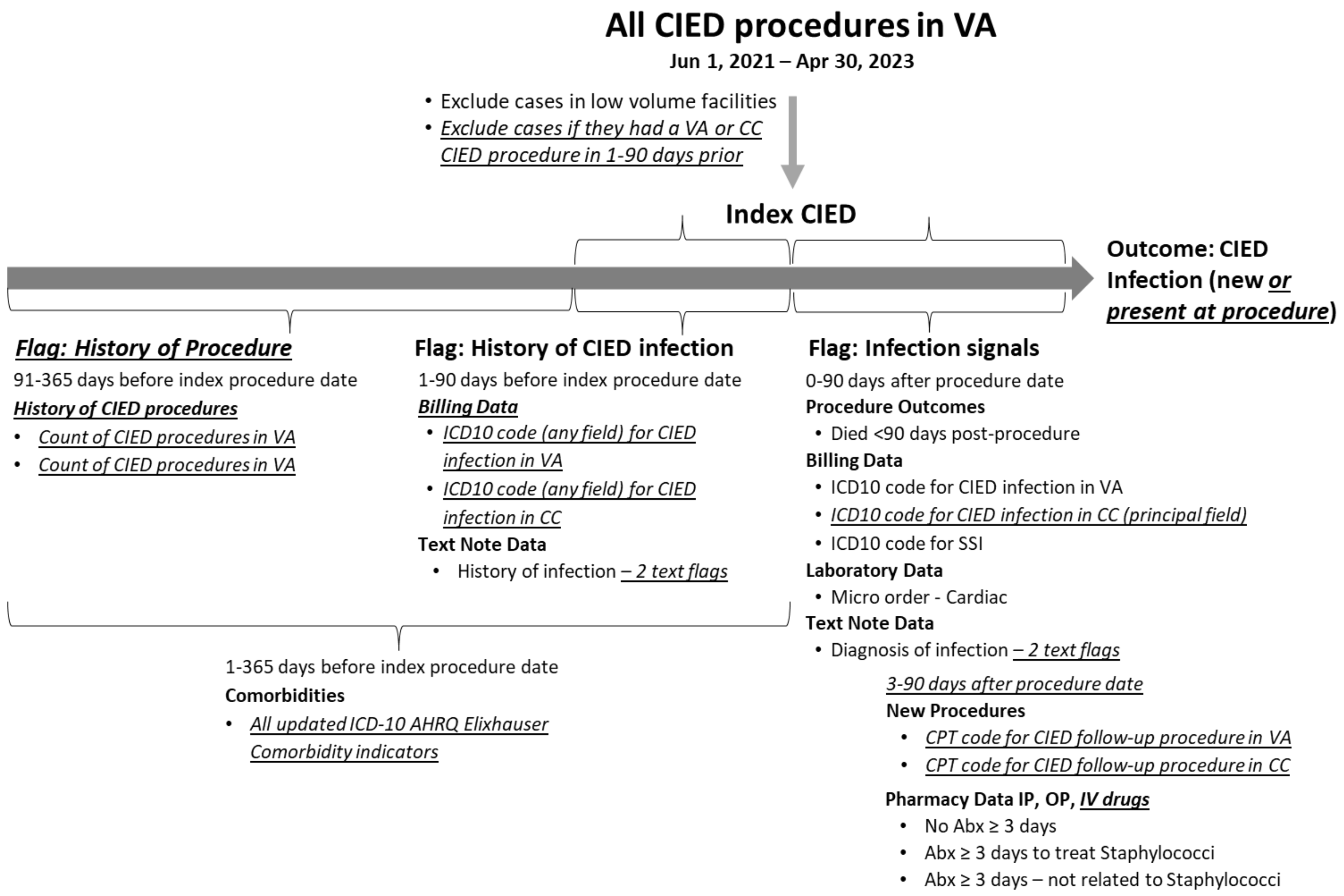

Italicized and underlined text indicate new potential flags and flag timing to identify index CIED procedures and 90-day infections. Plain text indicates original flags in algorithm. Grey arrow indicates time.

© 2025 Mull HJ et al. *JAMA Network Open*

eTable. Comparison of Changes in Timing for CIED Infection Prediction Flags from Original and Refined Algorithm

Original			Refined	
**Flag**	**Window (days)**		**Flag**	**Window (days)**
ICD10 CIED (VA)	(+) 3 → 90		ICD10 CIED (VA)	(+) 0 → 90
ICD10 SSI (w/o CIED) (VA)	(+) 3 → 90		ICD10 SSI (w/o CIED) (VA)	(+) 0 → 90
			ICD10 CIED (CC)	(+) 0 → 90
			Prior ICD10 (VA) CIED code	(-) 1 → -90
Micro cardiac orders	(+) 3 → 90		Micro cardiac orders	(+) 0 → 90
Text diagnosis of CIED infection	(+) 3 → 90		Text diagnosis of CIED “infect”	(+) 0 → 90
			Text diagnosis of “abscess”	(+) 0 → 90
Prior text diagnosis of CIED infection	(-) 3 → -90		Prior text diagnosis of CIED “infect”	(-) 1 → -90
			Prior text diagnosis of “abscess”	(-) 1 → -90
			CPT for CIED procedure (VA)	(+) 3 → 90
			CPT for CIED procedure (CC)	(+) 3 → 90
Antibiotics ≥ 3d	(+) 6 → 90		Antibiotics ≥ 3d	(+) 3 → 90
			Antibiotics ≥ 3d, include IVs	(+) 3 → 90
Antibiotics staph/no staph	(+) 6 → 90		Antibiotics staph/no staph	(+) 3 → 90
			Antibiotics staph yes/no, include IVs	(+) 3 → 90
Death in 90 days	(+) 0 → 90		Death in 90 days	(+) 0 → 90
			Death in 30 days	(+) 0 → 30
Comorbidities (Elix. Component beta ICD10)	(-) 1 → -365		Comorbidities (Elix. Component – ICD10 version)	(-) 1 → -365

© 2025 Mull HJ et al. *JAMA Network Open*

Notes: SSI=surgical site infections; CIED= cardiovascular implantable electronic device; CC=community care; CPT=Current Procedural Terminology; ICD=International Classification of Diseases Version 10; IV=intravenous; staph=*Staphylococcal*; Elix=Ellixhauser

eReferences

1. Noël PH, Copeland L. *Using VA Corporate Data Warehouse for Health Services Research*. 2012. *2012 VIReC Database and Methods Cyber Seminar Series*. http://www.hsrd.research.va.gov/for_researchers/cyber_seminars/archives/vdm-060412.pdf
2. Mull HJ, Stolzmann KL, Shin MH, Kalver E, Schweizer ML, Branch-Elliman W. Novel Method to Flag Cardiac Implantable Device Infections by Integrating Text Mining With Structured Data in the Veterans Health Administration's Electronic Medical Record. *JAMA Netw Open*. Sep 1 2020;3(9):e2012264. doi:10.1001/jamanetworkopen.2020.12264
3. VA Information Resource Center. Data Review Issue 8: Integrated Veteran Care (IVC) Consolidated Data Set (CDS). Accessed June 7, 2023. https://vaww.virec.research.va.gov/Reports/DR/DR-IVC-CDS.pdf
4. Agency for Healthcare Research and Quality (AHRQ). Elixhauser Comorbidity Software Refined for ICD-10-CM Healthcare Cost and Utilization Project (HCUP). Accessed Mar 21, 2023. www.hcup-us.ahrq.gov/toolssoftware/comorbidityicd10/comorbidity_icd10.jsp
5. Branch-Elliman W, Lamkin R, Shin M, et al. Promoting de-implementation of inappropriate antimicrobial use in cardiac device procedures by expanding audit and feedback: protocol for hybrid III type effectiveness/implementation quasi-experimental study. *Implement Sci*. Jan 29 2022;17(1):12. doi:10.1186/s13012-022-01186-8
6. CDC. Surgical Site Infection (SSI) Event. 2016. Updated January 2016. Accessed April 7, 2016. http://www.cdc.gov/nhsn/PDFs/pscManual/9pscSSIcurrent.pdf
7. Nyongesa D. Paper 4826-2020: Variable Selection Using Random Forests in SAS® Accessed Dec 1, 2022. https://support.sas.com/resources/papers/proceedings20/4826-2020.pdf
8. SAS Institute Inc. SAS/STAT® 9.2 User's Guide: The GLIMMIX Procedure. Feb 23, 2017. Accessed Sept. 1, 2016. https://support.sas.com/documentation/cdl/en/statugglmmix/61788/PDF/default/statugglmmix.pdf
9. SAS Institute Inc. SAS 9.2, Cary, NC, 2000-2004. 9.1 ed.

© 2025 Mull HJ et al. *JAMA Network Open*
