# Supplementary material for: Cardiac Implantable Device Infection Surveillance Algorithm
Source: JAMA Netw Open. 2025 Jun 5;8(6):e2514079. doi: 10.1001/jamanetworkopen.2025.14079 (PMC12142443; doi:10.1001/jamanetworkopen.2025.14079)
Supplement: Supplement 2. — Data Sharing Statement [file jamanetwopen-e2514079-s002.pdf]

Data Sharing Statement

Mull. Cardiac Implantable Device Infection Surveillance Algorithm. *JAMA Netw Open*. Published June 05, 2025. doi:10.1001/jamanetworkopen.2025.14079

Data
Data available: Yes
Data types: Other (please specify)
Additional Information: Data generated or analyzed during the study are available from the corresponding author by request and approval by the VA Privacy and Data Security Office.
How to access data: Data transfer will follow guidelines by the VA Privacy and Data Security Office.
When available: With publication

Supporting Documents
Document types: Statistical/analytic code
How to access documents: Statistical code is partially available in the Supplementary tables and full code is available from the corresponding author.
When available: With publication

Additional Information
Who can access the data: Anyone requesting the data following approval by the VA Privacy and Data Security Office.
Types of analyses: N/A
Mechanisms of data availability: Data access mechanism will be approved according to the guidelines of the VA Privacy and Data Security Office and depend on a variety of factors.
